# Hepatitis B Virus Reactivation in Gastrointestinal Stromal Tumor Patients Treated With Imatinib

**DOI:** 10.3389/fonc.2020.596500

**Published:** 2021-01-22

**Authors:** Tianxiang Lei, Fengbo Tan, Zhouhua Hou, Peng Liu, Xianhui Zhao, Heli Liu

**Affiliations:** ^1^Department of Gastrointestinal Surgery, Xiangya Hospital, Central South University, Changsha, China; ^2^Department of Infectious Diseases, Xiangya Hospital, Central South University, Changsha, China

**Keywords:** hepatitis B virus, reactivation, gastrointestinal stromal tumors, imatinib, mechanism

## Abstract

**Purpose:**

Hepatitis B virus reactivation (HBVr) in patients with gastrointestinal stromal tumors (GISTs) have not been sufficiently characterized. This study aimed to review the possible mechanism of HBVr induced by imatinib and explore appropriate measures for patient management and monitoring.

**Methods:**

The clinical data of GIST patients who experienced HBVr due to treatment with imatinib at Xiangya Hospital (Changsha, Hunan, China) were retrospectively analyzed. A literature review was also conducted.

**Results:**

Five cases were analyzed, including 3 cases in this study. The average age of the patients was 61.8 y, with male preponderance (4 of 5 vs. 1 of 5). These patients received imatinib as adjuvant treatment (n=4) or as neoadjuvant treatment (n=1). Primary tumors were mostly located in the stomach (n=4) or rectum (n=1). High (n=3) or intermediate (n=1) recurrence risk was categorized using the postoperative pathological results (n=4). Imatinib was then started at 400 (n=4) or 200 mg (n=1) daily. Patients first reported abnormal liver function during the 2^th^ (n=1),6^th^ (n=3), or 10^th^ (n=1) month of treatment with imatinib. Some patients (n=4) discontinued imatinib following HBVr; notably, 1 month after discontinuation, 1 patient experienced HBVr. Antivirals (entecavir n=4, tenofovir n=1), artificial extracorporeal liver support (n=1), and liver transplant (n=1) were effective approaches to treating HBVr. Most patients (n=3) showed favorable progress, 1 patient underwent treatment, and 1 patient died due to severe liver failure induced by HBVr.

**Conclusions:**

Although HBVr is a rare complication (6.12%), HBV screening should be conducted before starting treatment with imatinib in GIST patients. Prophylactic therapy for hepatitis B surface antigen positive patients, prompt antiviral treatment and cessation of imatinib are also necessary.

## Introduction

Gastrointestinal stromal tumors (GISTs) are the most common mesenchymal neoplasms located in the digestive tract, with an estimated annual incidence of 1–2 per 100,000 globally (1, 2). Most cases of GIST have activated mutations in KIT and platelet-derived growth receptor alpha (PDGFRA). Since 2000, imatinib has been found to target KIT changes in GIST cells (3). More than 80% of GIST patients have benefited from treatment with imatinib (4). This therapy has become one of the standard treatments for GIST (5).

Hepatitis B virus reactivation (HBVr) is a common complication in tumor patients with chronic HBV infection and simultaneously undergoing cytotoxic chemotherapy or immunosuppressive therapy (6). HBV is a major global health problem chronically affecting more than 257 million people worldwide (7). However, HBVr in tumor patients may lead to liver function injury or fatal liver failure. This occurrence can interrupt therapy, delaying the effective treatment of tumor patients and consequently affecting prognosis.

However, the risk for HBVr during the treatment of GISTs by using imatinib is poorly understood. To the best of our knowledge, only 2 cases have been reported worldwide (8, 9). Thus, it is urgent to clarify the mechanism and summarize such cases in order to draw attention from the medical community and provide clinical guidance for patient management.

## Methods and Materials

We retrospectively reviewed the data of 869 patients with GIST from January 2007 to June 2020 through the hospital information system of Xiangya Hospital of Central South University (Changsha, Hunan, China). A total of 440 patients received imatinib as adjuvant treatment (n=428) or neoadjuvant treatment (n=12); 49 patients tested positive for hepatitis B surface antigen (HBsAg), whereas 391 tested negative. Finally, 3 cases (6.12%) of HBVr due to imatinib for GIST were recorded. This study protocol was approved by the Medical Ethics Committee of Xiangya Hospital, Central South University (Changsha, Hunan, China).

## Results

### Case 1

A 50-year-old woman with a history of HBV infection over 20 years presented with abdominal pain, hematemesis, hematochezia, and even syncope on December 29, 2018. The patient had no experience using antivirals for HBV or hepatotoxic drugs. She was diagnosed with GIST by gastroscopy and abdominal-pelvic computed tomography (CT) scan. At the time of diagnosis, the patient had a normal liver function. Serologic tests for HBV showed that the patient was positive for HBsAg, hepatitis B e-antibody (HBeAb), hepatitis B core antigen (HBcAg) but negative for hepatitis B e-antigen (HBeAg) and hepatitis B surface antibody (HBsAb). The HBV DNA level was 3.10 × 10^4^ IU/mL (normal, <10 IU/mL). The patient underwent resection of two tumors with partial resection of the stomach on January 11, 2019. Postoperative pathology confirmed the diagnosis of two gastric GISTs [size, 10.5 cm × 7 cm × 6 cm and 4 cm × 2.5 cm × 2 cm; mitosis, both>5/50 high-power field (HPF)]. Immunohistochemistry indicated that the tumors were positive for CD34, CD117, Dog1, and Ki-67 (3%). Mutational analysis demonstrated a mutation in KIT exon 11. The patient was classified as high-risk under the modified National Institutes of Health (NIH) classification system (2008) (10). To reduce the risk of tumor recurrence, administration of imatinib 400 mg daily was started postoperatively in January 2019.

On June 25, 2019, the patient presented with nausea, vomiting, and jaundice. Liver function tests showed elevated aspartate transaminase (AST) at 434.0 U/L (normal, 13.0–35.0 U/L), alanine transaminase (ALT) at 407.0 U/L (normal, 7.0–40.0 U/L), total bilirubin at 24.5 umol/L (normal, 1.7–17.1 umol/L), and direct bilirubin at 12.4 umol/L (normal, 0.0–6.8 umol/L). No change in serum HBV serology was indicated, and the HBV DNA level increased to 3.57 × 10^6^ IU/mL. With the exclusion of other causes of liver injury such as infection with hepatitis A, C, or E virus, cytomegalovirus, Epstein-Barr virus, and autoimmune hepatitis, a diagnosis of imatinib-related chronic HBV reactivation was established. Thus, the patient immediately received entecavir (0.5 mg/d), discontinued imatinib, and took artificial extracorporeal liver support 3 times for severe liver damage. After 1 month, the symptoms and liver function abnormalities resolved. The HBV DNA level decreased to 6.59 × 10^2^ IU/mL. After discharge, the patient continued to take entecavir (0.5 mg/d) (up to present). In January 2020, the patient resumed taking low-dose imatinib (100 mg/d), with doses increased to 200 mg/d in February 2020 and 300 mg/d from March up to present. Patient recovery was uneventful. In April 2020, her liver function subsequently remained within normal ranges, No HBV DNA was detected, and no evidence of GIST recurrence was found in the abdominal–pelvic CT scan.

### Case 2

A 59-year-old man without any symptoms was diagnosed with GIST by physical examination including gastroscopy in November 2018. He was diagnosed with HBV infection 20 years ago without undergoing antiviral therapy. After admission to the local hospital, he tested positive for HBsAg, HBeAb, and HBcAg but negative for HBsAb, HBeAg, hepatitis C virus, and normal liver function. He underwent laparoscopic complete stomach resection of the tumor with an uncomplicated postoperative course. The mass was diagnosed as a gastric GIST and categorized as high-risk in accordance with the modified NIH classification system (2008) (10). (size, 7 cm × 5 cm × 4 cm mitosis, >5/50 HPF). Immunohistochemistry showed the following: CD117(+), CD34(+), Dog-1(+), and Ki-67 (<8%). Adjuvant treatment with imatinib (400 mg/d) was started on December 23, 2018.

In January 2019, the patient manifested abdominal pain. Laboratory and imaging tests showed normal results. The abdominal pain resolved after symptomatic treatment. In February 2019, the patient presented with edema of eyelids, hands, and ankles as side effects of imatinib. On May 15, 2019, the patient was admitted to a local hospital because of increasing systemic edema. Owing to serious side effects, imatinib administration was stopped. From November 2018 to May 2019, the patient received imatinib (400 mg/d), and his liver function was normal. No other hepatotoxic medication, except for imatinib, was taken.

However, after discontinuing imatinib, the patient reported dizziness, body weakness, and weight loss in June 2019. The patient was immediately admitted to Xiangya hospital. Laboratory findings showed the following measurements: ALT, 977.1 U/L (normal, 7.0–40.0 U/L); AST, 1382.0 U/L (normal, 13.0–35.0 U/L); total bilirubin, 20.5 umol/L (normal, 1.7–17.1 umol/L); and direct bilirubin, 11.3 umol/L (normal, 0.0–6.8 umol/L). Serology results indicated no change in serum HBV; moreover, HBV DNA was 6.11 × 10^7^ IU/mL (normal < 10 IU/mL), and the tumor marker CA125 was 82.73U/mL (normal, 0–35.00 U/mL). However, positron emission tomography–computed tomography did not indicate recurrence or metastasis of GIST and other tumors. Thus, HBVr was identified as the potential cause of liver damage, and the patient was immediately administered entecavir (0.5 mg/d). However, the clinical condition of the patient progressively deteriorated because of severe hepatitis caused by HBVr. The patient ultimately developed severe liver failure leading to death.

### Case 3

A 51-year-old man was found to have multiple hepatic masses and a gastric mass after a physical examination in September 2019. The patient subsequently underwent radical surgical resection for the gastric mass and palliative resection for the hepatic masses in a local hospital. The patient had a history of HBV infection of over 10 years but received no antiviral therapy for HBV. During hospitalization, the patient showed normal liver function, tested positive for HBsAg, HBeAb, and HBcAb, and tested negative for HBsAb, HBcAb, and hepatitis C virus. Pathological examination revealed that the gastric mass was a high-risk gastric GIST (size, 4.5 cm × 3.5 cm × 3 cm; mitotic index>10/50 HPF), which stained positive for CD117, CD34, DOG1, and ki-67 (20%). The liver mass was a metastatic GIST (the maximum tumor diameter was 1.5 cm), which stained positive for DOG1; one lymph node was metastatic (1/1). Additional molecular analysis confirmed the mutation in KIT exon 11. The liver metastases were only partly removed, requiring lifetime treatment with imatinib (400 mg/d), which the patient started to receive in January 2020.

Regular laboratory examination after treatment with imatinib for 3 months showed that the patient had mildly elevated ALT, 80.0 U/L (normal, 9.0–50.0 U/L), but no symptoms and signs. Laboratory examination after treatment with imatinib for 6 months showed further increases in ALT to 282.6 U/L (normal, 9.0–50.0 U/L), AST to 154.0 U/L (normal, 15.0–40.0 U/L), total bilirubin to 19.9 umol/L (normal, 1.7–17.1 umol/L), and direct bilirubin to 10.5 umol/L (normal, 0.0–6.8 umol/L). HBV DNA was 4.99 × 10^8^ IU/ml (normal, < 10 IU/ml). Serology testing results showed that HBsAg and HBcAb were positive, whereas HBsAb, HBeAb, and HBeAg were negative. After excluding other causes of hepatitis, we considered that hepatitis could be attributable to HBVr. Thus, entecavir (0.5 mg/d) was administered, and imatinib was discontinued immediately. The patient now was followed up under close observation.

## Literature Review

We performed a literature search in PubMed for other reported cases by using the terms “Hepatitis B virus reactivation”, “Gastrointestinal stromal tumors,” and “imatinib” and identified only 2 case reports (8, 9). The aforementioned cases, together with the 3 cases presented in the current study, complete the 5 cases currently reported (**Table 1**).

**Table 1:** Characteristics of cases with GIST who suffered HBV reactivation after receiving imatinib.

Author, year	Gender	Age (years)	Neoadjuvant or adjuvant treatment	Tumor type	Risk classification^a^	Dose of imatinib before HBVr(mg/d)	Time to onset of hepatic dysfunction from use of GIST (months)	HBV DNA before imatinib(IU/mL)	HBV DNA after HBVr (IU/mL)	Status of Imatinib following HBVr	Antiviral agent for HBVr (Dosage)	Liver transplant	artificial extracorporeal liver support	Outcome
Walker et al. (9)	M	62	Adjuvant treatment	Gastric GIST	Intermediate	400	6	NA	56600	Discontinued	Entecavir (NA)	Done	Not done	Recovered
Inayat et al. (8)	M	87	Neoadjuvant treatment	Rectal GIST	NA	200, held, 100, 400, held, 200	10	NA	397168540	Discontinued	Tenofovir (25 mg/d)	Not done	Not done	Recovered
Report (***Case 1***)	F	50	Adjuvant treatment	Gastric GIST	High	400	6	3.10×10^4^	3.57×10^6^	Discontinued	Entecavir (0.5 mg/d)	Not done	Done	Recovered
Report (***Case 2***)	M	59	Adjuvant treatment	Gastric GIST	High	400	6	NA	6.11×10^7^	Discontinued before HBVr	Entecavir (0.5 mg/d)	Not done	Not done	Died
Report (***Case 3***)	M	51	Adjuvant treatment	Gastric GIST and liver metastases	High	400	2	NA	4.99 x 10^8^	Discontinued	Entecavir (0.5 mg/d)	Not done	Not done	Under treatment

GIST, Gastrointestinal stromal tumor; ^a^the risk classification is based on the modified National Institutes of Health classification system (2008).

In the 5 cases, the average age of the patients was 61.8 y (range: 50–87 y), with a male-to-female ratio of 4:1. Four patients received imatinib as adjuvant treatment for intermediate—(n=1) or high-risk (n=3) recurrence based on the modified NIH classification system (2008) (10), and 1 patient received neoadjuvant treatment. Primary tumors were located in the stomach (n=4) or rectum (n=1), and one of our patients with a gastric GIST had liver metastasis. Imatinib at 400 mg daily was started in accordance with consensus-based medication (10) in 4 patients and 200 mg daily in 1 patient suffering from chronic kidney disease (stage 3b). Abnormal liver function was reported in 3 patients during the 6^th^ month of treatment with imatinib at 400 mg/d and in 1 patient during the 2^nd^ month of the same treatment at the same dose. Meanwhile 1 patient was diagnosed with HBVr during the 10^th^ month of treatment with 6 dose adjustments of imatinib, as follows: 200 mg/d for 10 d, held for 2 weeks; 100 mg/d for 3 months; and 400 mg/d for 40 d, held for 2 weeks, and restarted at 200 mg/d imatinib. We observed the change in HBV DNA (3.10 × 10^4^ IU/mL to 3.57 × 10^6^ IU/mL) before and after HBVr in 1 patient, which was attributed to treatment with imatinib, as well as the high level of HBV DNA following HBVr in 4 patients.

Treatment with imatinib was stopped in all patients following HBVr. Four patients received entecavir as antiviral treatment, and 1 patient received tenofovir. Two patients accepted liver transplant or artificial extracorporeal liver support to reverse the deterioration of their liver function. Three patients successfully recovered, 1 patient underwent treatment, and 1 patient died due to severe liver failure.

## Discussion

Imatinib has indeed changed the fate of patients with GIST and Philadelphia chromosome-positive chronic myeloid leukemia by targeting the oncogenic drivers of these diseases—BCR–ABL1 and KIT and/or PDGFRA—mutations that promote the function of tyrosine kinase activities (11). The importance of imatinib, as the mainstay of treatment for GIST, has been emphasized in various clinical guidelines (10, 12, 13). The side effects of imatinib are usually mild and generally well tolerated by most GIST patients (14). HBVr rarely occurs in patients infected with chronic HBV. However, when GIST patients with chronic HBV infection receive imatinib, the risk for HBVr is present. Without timely adequate recognition and treatment, HBVr can lead to an unfavorable prognosis or even death, such as that in *Case 2*, or increase the risk of tumor recurrence and progression, such as that in *Case 3*. GIST patients with chronic HBV infection and treated with imatinib rarely suffer from HBVr; regardless, these cases need to be consolidated to raise awareness of complications.

### Definition of HBVr

No unified diagnostic criteria for HBVr have been established worldwide; however, a consensus has been reached for patients with HBsAg(+)/HBcAb(-) or HBsAg(-)/HBcAb(+) receiving immunosuppressive therapy or chemotherapy. HBVr is defined as the occurrence of any of the following: a hundredfold increase in the HBV DNA level, the recurrence of detectable HBV DNA or positive HBsAg, or HBV DNA > 10^5^ IU/mL without a previously known HBV DNA level baseline (7, 15). Imatinib-induced hepatotoxicity has been reported and the incidence is less than 2.5% in patients with GIST (16); however, liver dysfunction usually resolves with either dose reduction or the discontinuation of the drug, which clearly contradicts *Case 2* in the current study. Thus, the 3 cases presented meet the definition of HBVr. Notably, HBVr occurs not only in patients with overt chronic HBV infection but also in patients with resolved HBV infection (17).

HBVr is usually divided into two stages: (i) immediately after immunosuppressive therapy with the induction of HBV replication, reflecting a sharp increase in serum HBV DNA, and (ii) during withdrawal or a decrease in immune suppression. In stage (ii), patients tend to develop immune reconstitution that may lead to hepatocellular injury and even liver failure (18).

### Mechanism of Imatinib-Induced HBVr

The mechanism by which imatinib induces HBVr has yet to be determined. However, a review of the relevant literature suggests that imatinib is directly and/or indirectly (by immunosuppressive function) involved in HBVr (**Table 2**). HBV covalently closed circular DNA and low levels of HBV DNA and RNA can be detected in host hepatocytes even in patients who have recovered from HBV and produced HBsAg after complete clearance of serum HBsAg and HBV DNA from a recent infection (26). When the immunity of the body, particularly the cellular immune function, is inhibited, viral replication increases, inducing an imbalance in the mechanisms that lead to HBVr (27). The imbalance between host immune response and HBV replication seem to indirectly cause HBVr. Although imatinib was not defined as an immunosuppressant, evidence has suggested that it exhibits an immunoregulatory effect. Imatinib at 400–800 mg/d inhibits the differentiation and function of dendritic cells (DCs), reducing the efficiency of priming cytotoxic T cell lymphocytes (CTLs) (19, 20). It also reduces memory B-cell frequencies and the secondary expansion of memory CTLs, resulting in impaired protection against reinfection (21, 22). Moreover, several *in vitro* studies using T cells isolated from human peripheral blood have demonstrated a dose-dependent reduction in T-cell proliferation and activation in the presence of imatinib (23, 24). Imatinib also inhibits immunity to several disease, such as herpes zoster infection (28), tuberculosis (29), Epstein Barr Virus-positive lymphoproliferative disease (30), and sclerotic-type chronic graft-versus-host disease, in addition to HBVr (31). However, imatinib can stimulate anticancer responses mediated by T cells, interferon-producing killer DCs and NK cells in patients (11, 32–34). Thus, further research is needed to distinguish the difference in effect of imatinib between viral immunity regulation and tumor immunity regulation.

**Table 2:** Mechanism of Hepatitis B virus reactivation induced by imatinib.

Reactivation modes of HBV	Activation mechanisms	Reference
Indirect reactivation of HBV	Inhibiting the differentiation and function of dendritic cells	(19, 20)
	Inhibiting memory cytotoxic T cell expansion	(21)
	Reducing memory B-cell and impairing humoral immune responses	(22)
	Reducing T-cell proliferation and activation	(23, 24)
Direct reactivation of HBV	Inhibiting c-Abl kinase to downregulate *CRL4* activity, suppress *CRL4^cdt2^*-mediated ubiquitination of HBV polymerase, and further promote HBV reactivation	(25)

In addition to indirect reactivation by immunosuppression, imatinib can directly activate HBV. Moreover, c-Abl kinase promotes the *CRL4^Cdt2^* mediated ubiquitination of HBV polymerase and further suppresses HBV replication. However, inhibiting c-Abl kinase activity with imatinib can lead to the accumulation of HBV polymerase protein and release of HBV, and consequently to HBVr (25). Clinical HBV replication in patients with chronic HBV may be activated *via* direct and indirect pathways; however the exact mechanism of HBVr induced by imatinib is unknown and needs further study.

### Prevention and Treatment

With the introduction of imatinib, GIST patients often have a good prognosis of long-term survival and quality of life. However, some patients develop HBVr, causing acute fatal liver failure and even death. According to the guideline (35), imatinib is associated with a moderate risk (1% to 10% incidence rate of HBVr). Therefore, early prevention, diagnosis, and treatment of HBVr are extremely significant for GIST patients receiving imatinib therapy.

Prompt identification of HBV-infected patients is mandatory before administering imatinib to prevent HBVr (36). HBV prophylaxis, identification of HBV patients, or reactivation monitoring can effectively prevent imatinib discontinuation. Conducting HBsAg and HBcAb tests is widely recommended before administering immunosuppressors (37, 38). If chronic HBV infection is evident, HBsAb, HBeAg, HBeAb, and HBV DNA may be tested selectively for risk stratification (39). HBsAg-positive tumor patients are suggested to undergo antiviral therapy 1 week before receiving immunosuppressors or are given antivirals and imatinib simultaneously and continue to suppress the risk for HBVr during treatment. The reason is that HBVr-induced hepatitis is more difficult to control and cure (37). Prophylactic therapy can potentially reduce the incidence of HBVr (40). ALT, AST and HBV DNA should be tested every 3 to 6 months during prophylaxis (6). Moreover, HBcAb-positive and HBsAg-negative GIST patients should be monitored every 1-3 months for their ALT, HBV DNA and HBsAg levels during treatment (6, 41). If the tests turn positive under close surveillance, antiviral treatment is started immediately (42). In principle, both therapy for HBV patients with normal immunity function and therapy for HBVr patients should reach the same therapeutic endpoint (43). GIST patients receiving antivirals during treatment with imatinib need to continue their antivirals for 6–12 months after treatment with imatinib (37, 44).

When GIST patients experience HBVr, treatment with imatinib should be stopped, and antiviral treatment should be immediately initiated to avoid unfavorable outcomes. As soon as HBVr was evident, imatinib was instantly discontinued in 4 (except *Case 2*) of the 5 cases presented. Most patients had a good prognosis. Owing to its efficacy in reducing the occurrence of HBVr, lamivudine is widely used in the treatment of cancer patients undergoin g chemotherapy (45). However, long-term use of lamivudine may lead to HBV resistance mutations and drug resistance. Compared with lamivudine, some more recent drugs such as entecavir may reduce the risk of drug resistance (46). Numerous studies (47, 48) have shown that entecavir can effectively inhibit viral replication and improve liver inflammation; moreover, it has an adequate safety index. Thus, entecavir is often considered as a first-line agent. Tenofovir disoproxil fumarate and tenofovir alafenamide fumarate also provide alternatives (49, 50).

One patient had artificial extracorporeal liver support and another patient had a liver transplant, and each had a good prognosis. Although some GIST patients with HBVr underwent antiviral and supportive treatment, their liver function still deteriorated. If liver injury could not be controlled by antivirals and liver-protective drugs, prompt artificial extracorporeal liver support as treatment choice would save the patient as in *Case 1*. For a favorable prognosis, a liver transplant is also a therapeutic choice (the case in Walker’s study) for patients with tumors (such as GIST) and suffering acute liver failure.

However, our study has several limitations. First, a small number of cases was included in the review, and the follow-up period was short. Second, this study is a retrospective study with possible selection bias. Third, the management and monitoring of HBVr in patients with GIST were not based on research concerning GIST patients with HBVr induced by imatinib but were merely suggested as feasible by current clinical evidence. More prospective clinical random control trials are needed to improve clinical understanding of severe complications.

## Conclusion

HBVr has a relatively low incidence rate (6.12%). Regardless, the occurrence of HBVr in GIST patients undergoing treatment with imatinib cannot be ignored, particularly in China, which has a high rate of HBV infection. HBVr prophylaxis and monitoring seem effective and safe for the management of these patients. Thus, serology testing for HBsAg and HBcAb in GIST patients and prophylactic therapy for patients at high risk for HBVr needs to be conducted prior to the initiation of treatment with imatinib. Meanwhile, cessation of treatment with imatinib and initiation of antiviral therapy are mandatory following HBVr.

## Data Availability Statement

The original contributions presented in the study are included in the article/supplementary material. Further inquiries can be directed to the corresponding author.

## Ethics Statement

The studies involving human participants were reviewed and approved by Xiangya Hospital of Central South University. The patients/participants provided their written informed consent to participate in this study. Written informed consent was obtained from the individual(s) for the publication of any potentially identifiable images or data included in this article.

## Author Contributions

TL was the lead investigator and contributed to writing the article. HL, FT, and ZH were the chief investigator and the senior author of the article. PL and XZ assisted with setting up the project and its promotion and helped with editing of the report. All authors contributed to the article and approved the submitted version.

## Funding

This study was supported by the Hunan Provincial Clinical Medical Technology Innovation Guidance Project, China (2018SK52604).

## Conflict of Interest

The authors declare that the research was conducted in the absence of any commercial or financial relationships that could be construed as a potential conflict of interest.

## Abbreviations

ALT, alanine transaminase; AST, aspartate transaminase; CT, computed tomography; GISTs, gastrointestinal stromal tumors; HBcAg, hepatitis B core antigen; HBeAb, hepatitis B e-antibody; HBeAg, hepatitis B e-antigen; HBsAb, hepatitis B surface antibody; HBsAg, hepatitis B surface antigen; HPF, high-power field; HBVr, hepatitis B virus reactivation; NIH, National Institutes of Health; PDGFRA, platelet-derived growth receptor alpha.
